# Topographic distribution of lymph node metastasis in patients with stage IB1 cervical cancer: an analysis of 8314 lymph nodes

**DOI:** 10.1186/s13014-021-01781-x

**Published:** 2021-03-20

**Authors:** Jing Cai, Xiaoqi He, Hongbo Wang, Weihong Dong, Yuan Zhang, Jing Zhao, Kay C. Willborn, Bangxing Huang, Zehua Wang, Ping Jiang

**Affiliations:** 1 Department of Obstetrics and Gynecology, Union Hospital, Tongji Medical College, Huazhong University of Science and Technology, 1277 Jiefang Avenue, Wuhan, 430022 China; 2 University Clinic for Medical Radiation Physics, Medical Campus Pius-Hospital, Carl von Ossietzky University Oldenburg, Georgstrasse 12, 26121 Oldenburg, Germany; 3 Department of Pathology, Union Hospital, Tongji Medical College, Huazhong University of Science and Technology, Wuhan, 430022 China

**Keywords:** Early-stage cervical cancer, Lymph node metastasis, Lymphadenectomy, Radiotherapy

## Abstract

**Background:**

Systematic pelvic lymphadenectomy or whole pelvic irradiation is recommended for the patients with stage IB1 cervical cancer. However, the precise pattern of lymphatic tumor spread in cervical cancer is unknown. In the present study we evaluated the distribution of nodal metastases in stage IB1 cervical cancer to explore the possibilities for tailoring cancer treatment.

**Methods:**

A total of 289 patients with cervical cancer of stage IB1, according to FIGO 2009, were retrospectively analyzed. All patients underwent laparoscopic radical hysterectomy (Querleu and Morrow type C2) and systematic pelvic lymphadenectomy with or without para-aortic lymphadenectomy (level 2 or level 3 according to Querleu and Morrow) from October 2014 to December 2017. Lymph nodes removed from 7 well-defined anatomical locations as well as other tissues were examined histopathologically, and typed, graded, and staged according to the WHO/IARC classification.

**Results:**

Totally 8314 lymph nodes were analyzed with the average number of 31.88 ± 10.34 (Mean ± SD) lymph nodes per patient. Nodal metastases were present in 44 patients (15.22%). The incidence of lymphatic spread to different anatomic sites ranged from 0% (presacral) to 30.92% (obturator nodes). Tumor size above 2 cm, histologically proven lymphovascular space involvement (LVSI) and parametrial invasion were shown to be significantly correlated with the higher risk of lymphatic metastasis, while obesity (BMI ≥ 25) was independently negatively associated with lymphatic metastases.

**Conclusions:**

The incidence of lymph node metastasis in patients with stage IB1 cervical cancer is low but prognostically relevant. Individual treatment could be considered for the selected low-risk patients who have smaller tumors and obesity and lack of the parametrial invasion or LVSI.

## Background

The incidence of lymph node metastasis in patients with stage IB1 cervical cancer has been reported to be 13–22% [1, 2]. Regardless of the low risk of lymph node metastasis, the whole pelvic chemoradiotherapy is often recommended [3, 4]. The common side effects related to the chemoradiotherapy of the total pelvic lymphatic system are lymphoedema and gastrointestinal toxicities [5–7]. Limiting the area of the treatment can reduce the treatment-related toxicities.

The precise pattern of lymphatic tumor spread of the early-stage cervical cancer is unknown. It is not clear how often lymph node metastases occur in different locations and which lymph nodes must be treated to completely cure all nodal metastatic deposits. The histology, tumor size and other risk factors of stage IB1 cervical cancer vary significantly and their influences on the lymphatic tumor spread are rarely known. More knowledge is required to set up a standard of a more patient oriented and risk-adapted treatment plan.

In this study we retrospectively examined the nodal metastases of stage IB1 cervical cancer and evaluated diverse risk factors for it, in term of to provide new insights into the clinical decisions in personalized treatment of early cervical cancer.

## Methods

### Samples

A total of 289 patients with stage IB1 cervical cancer, who underwent LRH and pelvic lymphadenectomies in our hospital from October 2014 to December 2017, were retrospectively analyzed, excluding the patients with previous treatments such as radiotherapy, chemotherapy or hormonal therapy. All cancers were pathologically confirmed. The presurgical staging examinations were performed using gynecological ultrasound, CT scan of the chest and abdomen and MRI of the pelvis. Distant metastasis were excluded. All removed lymph nodes were analyzed histopathologically in this study.

### Surgical procedure

The surgical procedures and techniques of LRH, pelvic lymphadenectomy and lymph node dissection were performed according to the C2 type of the Querleu-Morrow classification [8] and have been described previously [9]. All four surgeons have performed at least 100 LRH and pelvic lymphadenectomies.

Systematic bilateral pelvic lymphadenectomy includes the dissection of the common iliac, presacral, external iliac, internal iliac, and obturator lymph nodes. The dissection of parametric lymph nodes was a part of LRH. Para-aortic lymphadenectomy at aortic infra-mesenteric level was performed in the patients with highly suspective pelvic lymph nodes by visual inspection during surgery. All fatty and lymphatic tissues were removed completely in the specified area as follows:

### Anatomic landmarks of the specified area

- All precaval and preaortic lymph nodes the aortic bifurcations up to the level of inferior mesenteric artery were labeled as para-aortic lymph nodes.
- The common iliac lymph nodes lie between the bifurcation level of the aorta and the bifurcation of the iliac vessels.
- The lymph nodes situated along the external iliac vessels were labeled as external iliac lymph nodes; they include the lymph nodes caudal to the deep circumflex iliac vessels.
- All lymph nodes medial to the internal iliac vessel down to the level of the bifurcation of the uterine vessels were labeled as internal iliac lymph nodes.
- The presacral lymph nodes comprise of lymph nodes medial to the external iliac vessels, between internal and external iliac arteries, dorsal border is sacrum.
- The obturator lymph nodes lie between the external and internal iliac as the obturator lymph nodes, with the superior starting at the bifurcation of the internal and external iliac vessels and inferior at the obturator fossa.

### Histopathologic evaluation

Lymph nodes with metastatic cancer deposits from each anatomic location were histopathologically evaluated. All tumors were staged with the FIGO staging system [10] and typed/graded according to WHO/IARC Classification of Tumors [11].

### Statistical analysis

Statistical analyses were performed using SPSS 11.0 software package (SPSS Inc., Chicago, Illinois). The data were expressed as median and range or mean with standard deviation where appropriate. The rates of lymph node metastasis in the defined fields were shown as percentages. The differences in the lymph node metastasis rate between subgroups of patients stratified by clinicopathological characteristics were analyzed by χ2 tests. The risk factors for lymph node metastasis were identified by univariate and multivariate logistic regression analysis. The level of significance was set at 0.05 in all tests.

## Results

### Patients and tumor characteristics at the lymphadenectomies

In total, 289 patients were investigated in the present study. Median age of patients was 47 years (range 25–75). The mean body mass index (BMI) was 22.69 ± 3.19 kg/m^2^ (Mean ± SD, range: 16.66–25.53). The pathologic types of tumor were squamous cell cancer (n = 216 cases, 74.7%), adenocarcinoma (n = 53, 18.3%), adenosquamous carcinoma (n = 10, 2.6%), neuroendocrine tumor (n = 7, 7%) and one case (0.3%) each for SCC-Sarcoma-like carcinoma, SCC-Adenoid basal carcinoma and Yolk Sac tumor. Histologic grading showed a distribution of cases as follows: 31 were grade 1 (10.7%), 171 were grade 2 (59.2%) and 83 were grade 3 (28.7%).

### Distribution of excised lymph nodes

**Fig. 1:**
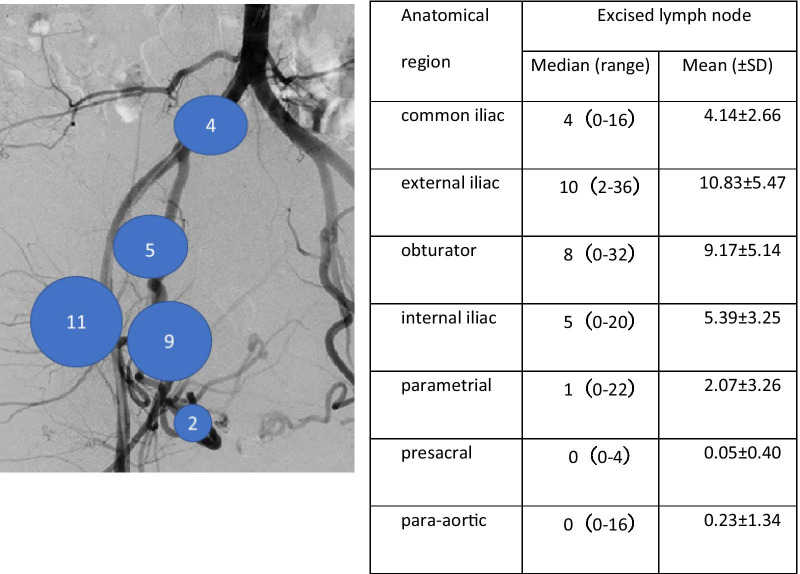
Average numbers of removed lymph nodes in each region

Totally 8314 lymph nodes were excised and mapped. On average 31.88 (± SD 10.34) lymph nodes were removed from each patient. Average numbers and anatomical regions of removed lymph node are showed in Fig. 1.

### Distribution of lymph nodal metastases

**Fig. 2:**
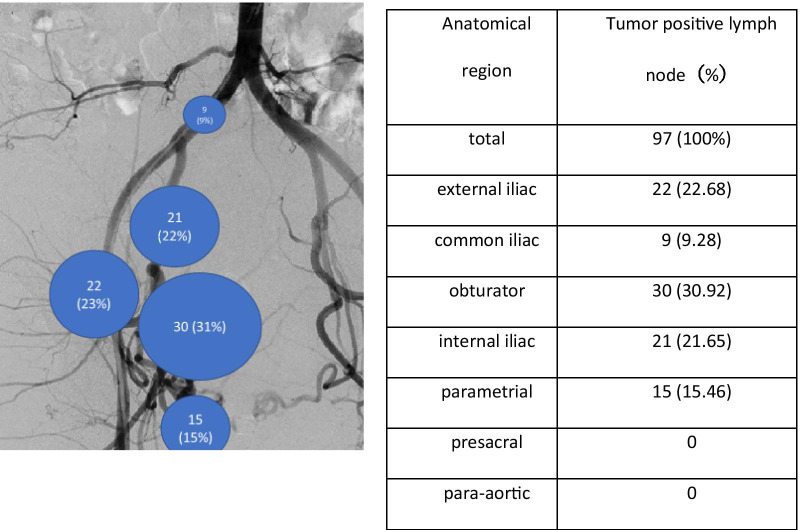
The distribution of lymph node metastasis in the patients with stage IB1 cervical cancer

There were 97 nodal metastasis presented in 44 (15%) of patients. The percentage of metastasis at different sites ranged from 0 (presacral, para-aortic nodes) to 30.92% (obturator nodes). The distribution of the lymph node metastasis counts from each region is shown in Fig. 2.

### Analysis of risk factors for the pelvic lymph node metastasis

The potential clinicopathologic risk factors associated with lymphatic metastasis were investigated and analyzed. 44 patients with node-positive disease, except one patient, had at least one known risk factor for lymph node metastasis.

As shown in Table 1, a significantly higher risk of lymph node metastasis was presented in patients with tumor size ≥ 2 cm (*p* = 0.001), deep stromal invasion (out 1/3 & middle 1/3 compare to inner 1/3, *p* = 0.012), LVSI (*p* < 0.001) and parametrial invasion (*p* < 0.001), while other factors like age, menopausal, pathological type and histological grading of cancer were not significantly correlated with LNM. Interestingly, our study showed that the risk of lymphatic metastasis was significantly reduced in obese patients (*p* = 0.025).

**Table 1:** Risk factors for lymph node metastases

Risk factors	Variable	Patients	Node positive (%)	*P* (χ^2^ test)
Age (years)	< 50	187	32 (17.11)	0.227
	≥ 50	102	12 (11.76)	
BMI (kg/m^2^)	≥ 25	52	1 (1.92)	0.025
	< 25	225	37 (16.44)	
	Unknown	12		
Menopausal	Premenopause	192	31 (16.15)	0.610
	Postmenopause	94	13 (13.83)	
Tumor size	< 2 cm	87	3 (3.45)	< 0.001
	≥ 2 cm	197	40 (20.30)	
Pathologic type	Squamous cell cancer	216	36 (16.67)	0.241
	Others	73	8 (10.96)	
Histologic grading	< G2	31	2 (6.45)	0.229
	≥ G2	254	42 (16.54)	
	< G3	202	28 (13.86)	0.250
	≥ G3	83	16 (19.28)	
Parametrium invasion	Absent	265	32 (12.08)	< 0.001
	Present	24 (8.3%)	12 (50.00)	
Stromal invasion	Inner one-third	126	11 (8.73)	0.012
	Middle one-third	72	14 (19.44)	
	Outer one-third	82	19 (23.17)	
Stromal invasion	Inner two-third	209	25 (11.96)	0.018
	Outer one-third	82	19 (23.17)	
Lymphovascular space involvement (LVSI)	Absent	192	17 (8.85)	< 0.001
	Present	97 (33.6)	27 (27.84)	
Removed lymph nodes	< 30	144	24 (16.67)	0.497
	≥ 30	145	20 (13.79)	

To further confirm the identified risk factors, the multivariate logistic regression analysis were performed (Table 2). Only positive parametrial extension, tumor diameter of 2 cm or greater and LVSI were associated with lymph node metastasis. Obesity (BMI ≥ 25) was independently significantly negatively correlated with the risk of lymphatic metastases.

**Table 2:** Risk factors of lymph node metastasis, univariate analysis and multivariate analysis

Variable	Univariate analysis			Multivariate analysis		
	OR	95% CI	*P* value	OR	95% CI	*P*
BMI ≥ 25	0.100	0.013–0.744	0.025	0.057	0.007–0.479	0.008
Tumor size ≥ 2 cm	7.134	2.14–23.75	0.001	4.350	1.197–15.816	0.026
Positive parametrial extension	7.28	3.02–17.58	< 0.001	8.448	2.487–28.693	0.001
Outer one-third invasion of stroma	2.292	1.18–4.45	0.014	0.948	0.311–2.886	0.925
Presence of LVSI	3.971	2.04–7.74	< 0.001	1.965	0.821–4.704	0.129

*OR* odds ratio, *CI* confidence interval

### The survival outcomes of patients with the risk factors

Postoperative radiotherapy was recommended to the patients with positive margin or lymph node metastasis or parametrial involvement, as well as to the patients with at least 2 of the 4 followed risk factors: LVSI, tumor size > 4 cm, deep stromal invasion, and adenocarcinoma. Follow-up examinations were performed in intervals of 3 months in the first year and in six-months-intervals thereafter up to 10 years. Median follow-up was 50 months (Range: 14–67 months).

**Fig. 3:**
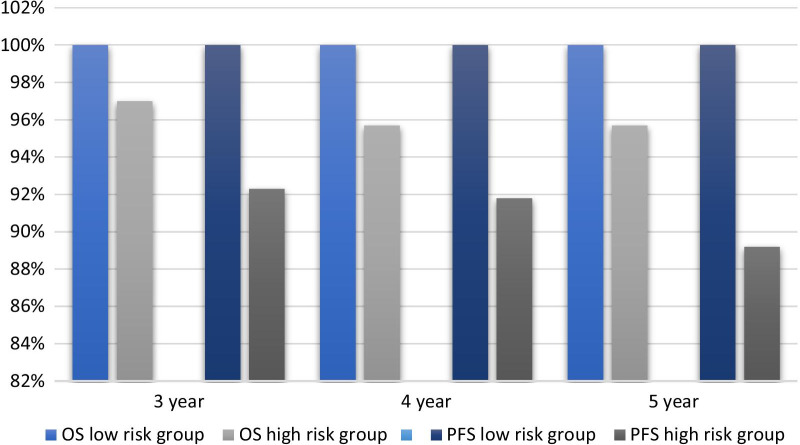
The survival outcomes of patients with the significant risk factors. High-risk group: tumors size ≥ 2 cm or BMI < 22.5 kg/m^2^ or present of the parametrial invasion or LVSI. Low-risk group: tumors size < 2 cm and BMI ≥ 22.5 kg/m^2^ and absent of the parametrial invasion or LVSI

We analyzed and compared the survival outcomes of patients with different risk factors. As shown in Fig. 3, patients with tumors size < 2 cm, BMI ≥ 22.5 kg/m^2^ and lack of parametrial invasion or LVSI were defined as low-risk group. A 100% 5 year-Progression-free survival (PFS) and 100% 5 year-overall survival (OS) were observed in the low-risk group. In contrast, the 5 year-PFS rate and 5 year-OS rate in the high-risk group were 89.2% and 95.7%, respectively.

**Fig. 4:**
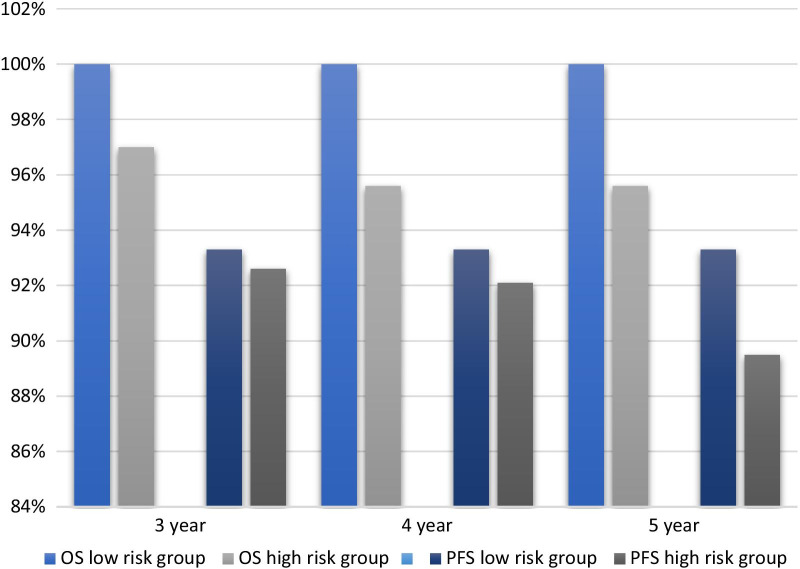
The survival outcomes of patients with the preoperatively assessable risk factors. High-risk group: tumors size ≥ 2 cm or BMI < 22.5 kg/m^2^. Low-risk group: tumors size < 2 cm and BMI ≥ 22.5 kg/m^2^

We thereafter analyzed the patients with preoperatively assessable risk factors and defined patients with tumors size < 2 cm and BMI ≥ 22.5 kg/m^2^ as low-risk group. One case of relapse was confirmed in this group. As shown in Fig. 4, the 5 year-PFS rate in the low-risk group was 93.3% and the 5 year-OS rate was 100%. The 5 year-PFS rate and OS rate in the high-risk group with were 89.5% and 95.6%.

### Factors associated with lymph node metastases in different anatomical region

We next further investigated the association of risk factors with lymphatic metastasis to specific anatomical locations. The multivariate regression analysis showed that the presence of parametrial invasion significantly increases the risk of lymph node metastases (LNM) in each location. The odds ratio for the existence of external iliac LNM is at over 10 (*p* < 0.001). Histologically proven LVSI correlated independently significantly with the higher risk of LNM of the external iliac, obturator and the parametrial areas. Notably, tumor larger than 2 cm showed no predictive value for regionally specific metastasis (Table 3).

**Table 3:** Multivariate analysis of risk factors associated with lymph node metastasis in different anatomical region

	Parametrium invasion			Stromal invasion > 2/3			LVSI			Tumor size > 2 cm			LNM of common iliac			
	OR	95% CI	*P*	OR	95% CI	*P*	OR	95% CI	*P*	OR	95% CI	*P*	OR		95% CI	*P*
Common iliac	5.932	1.028–34.213	0.046	2.675	0.529–13.540	0.234	0.989	0.178–5.499	0.990	–	–	0.997	–		–	–
External iliac	10.500	3.553–31.027	< 0.001	4.122	1.511–11.244	0.006	5.280	1.803–15.460	0.002	3.503	0.783–15.662	0.101	12.682		1.469–109.465	0.021
Obturator	6.863	2.468–19.086	< 0.001	1.240	0.486–3.164	0.652	3.880	1.567–9.604	0.003	–	–	0.996	58.636		5.275–651.800	0.001
Internal iliac	4.217	1.245–14.279	0.021	2.131	0.766–5.930	0.147	2.067	0.751–5.688	0.160	7.088	0.921–54.533	0.060	5.888		0.771–44.982	0.087
Parametrial	6.167	1.439–26.434	0.014	2.147	0.562–8.208	0.264	7.389	1.505–36.282	0.014	3.640	0.448–29.561	0.227				

*LNM* lymph node metastasis, *OR* odds ratio, *CI* confidence interval

## Discussion

Our study investigated a large homogenic samples of patients with the stage IB1 cervical cancers and showed an incidence of lymph node metastasis at 15% of patients. This result is consistent with the previously published data from smaller series, which mostly comprise of the heterogenic sample of early-stage cervical cancer [1, 2, 12, 13]. The standard treatment aiming at the total pelvic lymphatic system seems to be unnecessary to majority of the patients.

Subdividing patients for personalized treatment based on high-risk features minimize treatment and its morbidity has attracted more and more attention. Togami et al. investigated patients with stage IA2-IIB cervical cancers and reported that parametrial involvement and primary tumor size greater than 2 cm increased the risk of pelvic lymph node metastasis, which implicates the importance of tailoring the treatment for the low-risk patients [14]. In our study, tumor size ≥ 2 cm, BMI < 25 kg/m^2^, the parametrial invasion and LVSI are shown to increase the risk of the lymphatic metastasis significantly and independently. Among all 44 patients with lymph node metastasis, there is only one patient (2.2%) without any risk factors. The patients with tumors size < 2 cm and BMI ≥ 22.5 kg/m^2^ and lack of parametrial invasion or LVSI were defined as low-risk group and in the low- risk group. A 100% 5 year-OS and 100% 5 year-PFS were observed in the low-risk group. That is, relapse was not observed, even if there was only one lymph node metastasis found and removed from all the patients in the low-risk group. The number of lymph nodes, that must be treated, should be further discussed. In contrast, the 5 year-OS rate and 5 year-PFS rate in the high-risk group were 95.7% and 89.2%, respectively. We analyzed separately the survival outcomes in the patients with tumors size < 2 cm and BMI ≥ 22.5 kg/m^2^, which preoperatively assessable are. One case of relapse was confirmed. The 5 year-OS rate in the low-risk group was 100% and the 5 year-PFS rate was 93.3%. The 5 year-OS rate and 5 year PFS rate in the high-risk group with were 95.6% and 89.5%. Therefore, we strongly suggested, that the whole pelvic chemoradiotherapy or radical lymphadenectomy should be well discussed before recommended to the low-risk patients because an adjuvant or salvage therapy is still possible.

Recently, the safety and the feasibility of the less radical surgery in early stage IB1 cervical cancer were evaluated in three ongoing studies: Concerv, GOG-278 and SHAPE [16, 17]. Tumors with low risk, such as tumor size < 2 cm e.g. were treated with non-radical hysterectomy to avoid the bladder, bowl, and sexual dysfunction and therefore improve the life quality. However, pelvic lymph node dissection is still suggested to the patients with the low-risk cancer, with the exception of Concerv study, in which lymphatic mapping with sentinel lymph node biopsy are allowed. Other than identifying the risk factors for lymph node metastasis in early-stage cervical cancer, our study further evaluated the predictive value of the risk factors in different anatomic location and provided more evidence for the less radical treatment of the lymphatic system in early-stage cervical cancer, which would reduce treatment related toxicities and benefit the patients. Further investigation and more randomized prospective clinic studies are strongly required.

Buchsbaum et al. described three routes of lymphatic flow of the uterine cervix [18]. The most common route is the lateral channel to the obturator, internal iliac and common iliac nodes. The second path anteriorly terminates in the external iliac. And the rarest route reaches posteriorly to the sacral, common iliac and para-aortic nodes. Our results showed over 80% lymph node metastasis of stage IB1 cervical cancer were found in obturator, external iliac, internal iliac, parametric lymph nodes, which clearly support his theory. Skipping low risk locations such as common iliac will reduce the treatment-related toxicities for selected patients. The treatment of the presacral lymph nodes or inguinal lymph node should be carefully considered. When severe side effects occur during the radiotherapy, focusing on the high-risk locations could be an alternative to the termination of the treatment.

It has been shown that adjuvant pelvic radiotherapy significantly improved the progress free survive for the patient with “intermediate-risk” disease [19], whereas the adjuvant pelvic chemoradiation significantly improved the overall survive for the patients with “high-risk” disease [20]. The National Comprehensive Cancer Network (NCCN) suggests in Clinical Practice Guidelines 2013 that the postoperative radiation area should include the parametria, presacral, obturator, internal iliac, and external iliac nodal regions in cervical cancer [21]. There is no further change in the latest version 2019. In our study there was no lymph node metastasis founded in the presacral region. We demonstrate that, the risk of lymph node metastases in all areas will be increased if there is a parametrial invasion with pathological evidence. The most common metastases acquired at extranal iliac lymph nodes. The LVSI of cervical cancer is also highly related with lymphatic metastases in the external iliac, obturator and parametrial areas, among which the parametrial lymphatic metastasis has highest frequency. If there is a common iliac lymphatic metastasis, patients will obtain more than 59-times higher chances to have the obturator LNM. So for the individualized radiotherapeutic strategy considering such risk factors for the patients with early stage cervical cancer will reduce certain side effects and increase the efficacy of target treatment.

Interestingly, obesity (BMI ≥ 25) was showed in our study as an independent negative predictor of lymphatic metastases. Li et al. reported that the age of patients old than 40 year was a protective factor for lymph node metastases for the cervical patients [22]. However, we show that age ≥ 50 is not correlated with the risk of lymph node metastases. More investigation is required to understand how the biological characteristics of the patients influence the disease process.

The technique protocols of the laparoscopic radical hysterectomies and pelvic lymphadenectomies in our hospital are well defined and standardized. In our study the average number of lymph nodes removed from each patient was 32. All patients underwent an adequate lymphadenectomy, given that the retrieval of a total number of 20 pelvic lymph nodes is the gold standard for adequate complete lymphadenectomy [23]. Previously we reported a 3-year overall survival rate of 94.9% and the 3-year progression-free survival rates were 94.1% for patients with stage IB1 cervical cancer using this technique [9]. Lots of other studies also have showed the equal efficacy of the laparoscopic radical hysterectomies and pelvic lymphadenectomies [24–26]. A recently published prospective study observed survival benefits with the classic laparotomy in early cervical cancer and a fourfold higher local recurrence rate in the laparoscopic arm, in our opinion perhaps due to the abdominal access to excise the tumor instead of the widely used vaginal access in the laparoscopic arm [27].

Our study is a retrospective design. Due to the lack of prospective data, the use of sentinel lymph node biopsy was not included in the present study. Marinitz et al. reported that in patients with stage 1B1/1B2 cervical cancer, the sentinel lymph nodes sites are: 3% at common iliac external iliac, 67% at interiliac, 9% at internal iliac and 8% at parametric [28]. This discrepancy with our findings on the distribution of lymph node metastasis needs further investigations in the clinic.

## Conclusions

Despite the limitations of our study, we confirmed four significant and independent risk factors for the lymph node metastasis in early-stage cervical cancer, which can be used to identify the low-risk group patients and therefore the potential candidates for less radical treatment.

## Data Availability

The datasets used and/or analyzed during the current study are available from the corresponding author on reasonable request.

## Abbreviations

LVSI: Lymphovascular space involvement
LRH: Laparoscopic radical hysterectomies
BMI: Body mass index
LNM: Lymph node metastasis
PFS: Progression-free survival
OS: Overall survival

## References

[1] Zhou J, Ran J, He ZY (2010). Tailoring pelvic lymphadenectomy for patients with stage IA2, IB1, and IIA1 uterine cervical cancer. GynecolOncol.

[2] Kim MK, Kim JW, Kim MA (2010). Feasibility of less radical surgery for superficially invasive carcinoma of the cervix. GynecolOncol.

[3] Gray HJ (2008). Primary management of early stage cervical cancer (IA1-IB) and appropriate selection of adjuvant therapy. J NatlCompr Cancer Netw.

[4] Salicrú SR, de la Torre JF, Gil-Moreno A (2013). The surgical management of early-stage cervical cancer. CurrOpinObstetGynecol.

[5] Abu-Rustum NR, Alektiar K, Iasonos A (2006). The incidence of symptomatic lower-extremity lymphedema following treatment of uterine corpus malignancies: a 12-year experience at Memorial Sloan-Kettering Cancer Center. GynecolOncol.

[6] Le Borgne G, Mercier M, Woronoff AS (2013). Quality of life in long-term cervical cancer survivors: a population-based study. GynecolOncol.

[7] Perez CA, Grigsby PW, Lockett MA (1999). Radiation therapy morbidity in carcinoma of the uterine cervix: dosimetric and clinical correlation. J Int J RadiatOncolBiolPhys.

[8] Querleu D, Morrow CP (2008). Classification of radical hysterectomy. Lancet Oncol.

[9] Yang L, Cai J, Dong W, Shen Y (2015). Laparoscopic radical hysterectomy and pelvic lymphadenectomy can be routinely used for treatment of early-stage cervical cancer: a single-institute experience with 404 patients. J Minim Invasive Gynecol.

[10] FIGO Committee on Gynecologic Oncology (2014). FIGO staging for carcinoma of the vulva, cervix, and corpus uteri. Int J GynaecolObstet Off Organ Int Fed GynaecolObstet.

[11] Kurman RJ, Carcangiu ML, Herrington CS (2014). WHO/IARC classification of tumours of female reproductive organs.

[12] Zhou J, Ran J, He ZY (2015). Tailoring pelvic lymphadenectomy for patients with stage IA2, IB1, and IIA1 uterine cervical cancer. J Cancer.

[13] Lee JM, Lee KB, Lee SK (2007). Pattern of lymph node metastasis and the optimal extent of pelvic lymphadenectomy in FIGO stage IB cervical cancer. J ObstetGynaecol Res.

[14] Togami S, Kamio M, Yanazume S (2014). Can pelvic lymphadenectomy be omitted in stage IA2 to IIB uterine cervical cancer?. Int J Gynecol Cancer.

[15] MD Anderson Cancer Center. Conservative surgery for women with cervical cancer. http://clinicaltrials.gov/show/NCT01048853. NLM Identifier: NCT 01048853.

[16] Covens A. GOG Protocol 278. http://www.gcig.igcs.org/Spring2012/2012_june_cervix_cancer_committee.pdf.

[17] Plante M. The SHAPE trial. http://www.gcig.igcs.org/Spring2012/2012_june_shape_trial.pd.

[18] Buchsbaum HJ (1979). Extrapelvic lymph node metastasis in cervical carcinoma. Am J ObstetGynecol.

[19] Rotman M, Sedlis A, Piedmonte MR (2006). A phase III randomized trial of postoperative pelvic irradiation in stage IB cervical carcinoma with poor prognostic features: follow-up of a gynecologic oncology group study. Int J RadiatOncolBiolPhys.

[20] Peters WA, Liu PY, Barrett RJ (2000). Concurrent chemotherapy and pelvic radiation therapy compared with pelvic radiation therapy alone as adjuvant therapy after radical surgery in high-risk early-stage cancer of the cervix. J ClinOncol.

[21] Koh WJ, Greer BE, Abu-Rustum NR, Apte SM, Campos SM, Chan J (2013). Cervical cancer. J NatlCompr Cancer Netw.

[22] Li X, Yin Y, Sheng X, Han X (2015). Distribution pattern of lymph node metastases and its implication in individualized radiotherapeutic clinical target volume delineation of regional lymph nodes in patients with stage IA to IIA cervical cancer. RadiatOncol.

[23] Panici BP, Scambia G, Baiocchi G (1991). Technique and feasibility of radical para-aortic and pelvic lymphadenectomy for gynecologic malignancies: a prospective study. Int J Gynecol Cancer.

[24] Lee EJ, Kang H, Kim DH (2011). A comparative study of laparoscopic radical hysterectomy with radical abdominal hysterectomy for early-stage cervical cancer: a long-term follow-up study. Eur J ObstetGynecolReprodBiol.

[25] Nam JH, Park JY, Kim DY (2012). Laparoscopic versus open radical hysterectomy in early-stage cervical cancer: long-term survival outcomes in a matched cohort study. Ann Oncol.

[26] Malzoni M, Tinelli R, Cosentino F (2009). Laparoscopic radical hysterectomy with lymphadenectomy in patients with early cervical cancer: our instruments and technique. SurgOncol.

[27] Ramirez PT, Frumovitz M, Pareja R (2018). Minimally invasive versus abdominal radical hysterectomy for cervical cancer. N Engl J Med.

[28] Marnitz S, Köhler C, Bongardt S, Braig U, Hertel H, Schneider A, German Association of Gynecologic Oncologists (AGO) (2006). Topographic distribution of sentinel lymph nodes in patients with cervical cancer. GynecolOncol.

